# Simple new technique for macular pucker peel without forceps

**DOI:** 10.3389/fmed.2022.947578

**Published:** 2022-09-13

**Authors:** Xhevat Lumi, Beáta Eva Petrovski, Goran Petrovski

**Affiliations:** ^1^Eye Hospital, University Medical Center Ljubljana, Ljubljana, Slovenia; ^2^Center for Eye Research, Department of Ophthalmology, Oslo University Hospital, Oslo, Norway; ^3^Department of Ophthalmology, Institute for Clinical Medicine, Faculty of Medicine, University of Oslo, Oslo, Norway; ^4^Department of Ophthalmology, University of Split School of Medicine and University Hospital Center, Split, Croatia

**Keywords:** epiretinal membrane, macular pucker peel, pars plana vitrectomy, best corrected visual acuity, central retinal thickness

## Abstract

**Purpose:**

This study aimed to describe the effectiveness and evaluate the anatomical and functional results of surgery for macular pucker (MP) peel using a 25-gauge pars plana vitrectomy (PPV) cutter without forceps.

**Methods:**

This study assessed a prospective consecutive case series of 14 eyes of 14 patients who underwent 25-gauge PPV for MP. The surgical procedure was performed using the new peeling technique. The edge of the membrane was engaged at the opening of the cutter by gradually increasing the vacuum. The peeling process was finished by holding a stable vacuum or regrasping the membrane in the same manner.

**Results:**

The study included six women and eight men patients with a mean age of 72.3 (range 59–84) years. MP peel was achieved in all cases without the need for microforceps. Patients were followed for at least 6 months. Visual acuity and retinal thickness were obtained 6 months after the surgery. Best corrected visual acuity improved from a mean pre-operative 0.6 on a logMAR scale to post-operative 0.23 (*P* < 0.001). Mean pre-operative Central Retinal Thickness was significantly reduced from 489.7 to 377.6 μm post-operatively (*P* < 0.001). There were no intra- or post-operative complications.

**Conclusion:**

MP peel with a 25-gauge vitrectomy probe could be an alternative simple and safe technique. The technique does not require extra instrumentation. It results in anatomic and functional improvement in all cases.

## Introduction

Epiretinal membranes (ERM) are a collection of cells and an extracellular matrix that grow on the inner surface of the retina, mainly localized on the central retina or the macula lutea (1). In the optical coherence tomography (OCT) definition by Hubschman et al., the ERM is considered to be an irregular and hyperreflective layer over the inner limiting membrane (ILM) frequently associated with the presence of hyporeflective spaces between the ERM and the ILM, and with signs of wrinkling of the underlying retina (2).

ERMs may be located peripherally and have little or not any impact on visual function (3). More commonly, they have central foveal or perifoveal localization leading to significant visual impairment and worsening the quality of life (4). The prevalence of ERM increases with age (5). Most often, ERMs occur in individuals older than 50 years, approaching the incidence of 20% of the total population by the age of 70 years (4).

There are different classification schemes of ERMs, but in general, more traction on the fovea causes more disruption to the macular anatomy, more severe visual acuity impairment, and more symptoms like reduced visual acuity, blurred vision, metamorphopsia, loss of stereopsis, and aniseikonia (6).

Gass JDM was the first to propose a clinical classification of ERMs based on the ophthalmoscopic appearance and distortion of the central retina by the membrane (7). Despite modern classifications with various OCT classification schemes, in clinical practice, the most often used is the two-stage classification scheme, which categorizes the ERMs as *cellophane maculopathy* (with an early, translucent form of ERM without distortion of the inner retina) and *macular pucker* (MP; with a late, opaque form of ERM having a distortion of the inner retina) (6, 8–11).

Currently, there are no medical treatment options for MP. The management consists of either observation or surgical intervention. The modern surgical approach for treating MP consists of sutureless transconjunctival three-port (23-, 25-, or 27- gauge) pars plana vitrectomy (PPV) and peeling of the ERM and/or ILM usually performed under a higher magnification lens (6). ERM peeling usually starts with a “pinch and peel” technique by using ILM or any other microforceps. Beside ILM forceps, there are other tools that can be used either to start or to complete the peeling, including micro-vitreoretinal blades, needle picks, diamond-dusted scrapers, and flex loops (12). Since multiple attempts in order to engage and peel the membrane are necessary, iatrogenic damage by the instrumentation may happen. Unintentional and accidental pinches to the nerve fiber layer and contusional retinal pigment epithelial cells lesions as well as multiple focal retinal hemorrhages have also been described (12).

Herein, we report a simple, cost- and time-saving technique for MP peeling without using micro-forceps or any other instrumentation except the simplest standard PPV tools.

## Subjects and methods

Fourteen patients having MP who underwent surgery from May 2020 to August 2021 at the Eye Hospital, University Medical Center Ljubljana, Slovenia, were included in the study. The study design was prospective interventional non-comparative case series. Enrolled were only patients with idiopathic MP in which membrane peeling was performed using only a vitreous cutter. Patients with a history of other ocular diseases, macular diseases, retinal dystrophies, previous vitreoretinal surgeries, and myopia of more than 5 diopters were excluded. The indication for surgery in each of the cases was decreased visual acuity and metamorphopsia.

Prior to surgery, all patients underwent a complete ophthalmological examination including Snellen best corrected visual acuity (BCVA), intraocular pressure measurement (IOP), slit lamp anterior segment examination, and dilated funduscopic examination. The Snellen acuity was converted into a logarithm of the minimum angle of resolution (logMAR) equivalent. Optical Coherence Tomography (Swept-source OCT, DRI OCT Triton, Topcon) was performed and used for evaluation of the macular area, MP edges, and morphological structural changes on the retinal layers. The following pre- and post-operative parameters were analyzed on OCT: central retinal thickness (CRT), presence of ERM, presence of macular hole (MH), continuity or discontinuity of external limiting membrane (ELM), ellipsoid zone (EZ), and retinal pigment epithelium (RPE), as well as presence or absence of subretinal fluid (SF). We considered the following primary outcomes: mean change in BCVA in the studied eye between baseline and 6 months after the surgery, change in the mean CRT, and complications related to the surgical technique. Secondary outcomes were the following: recurrence of ERM during the follow-up period, MH occurrence, post-operative ELM, EZ, and RPE continuity, and presence of SF.

The study was approved by the National Medical Ethics Committee of the Republic of Slovenia and adhered to the tenets of the Declaration of Helsinki. Informed consent was obtained from all patients before surgery and all of them had a proper follow-up.

### Surgical technique

In all cases, the surgical technique involved a 25-gauge three-port PPV. Surgeries were performed with the Constellation platform (Alcon Laboratories, Fort Worth, TX, USA) under sub-tenon's anesthesia. In cases with nuclear sclerosis or significant cataract, phacoemulsification with single piece IOL in-the-bag implantation was carried out together with vitrectomy. In all patients, Brilliant Peel Dual Dye (Fluoron GmbH, Germany) was used for staining the ERM. The proper edge of the ERM was first identified. After completing the vitrectomy, the opening of the 25-gauge cutter was oriented toward the edge of the MP. The cutting function was turned off from the foot pedal. By stepping gently on the pedal and increasing gradually the vacuum, it was possible to see the engaged edge of the membrane on the cutter. The proper engagement was achieved at a 300 mmHg vacuum. By holding the stable vacuum, we started to elevate the edge of the membrane. In case the vacuum level was not sufficient to continue peeling, the aspiration was gradually increased up to 500 mmHg. Once the edge was elevated, then stable vacuum was maintained, while the membrane was carefully elevated. During this maneuver, several times it was necessary to release the edge of the membrane by stopping the aspiration on the foot pedal or activating the reflux with the foot pedal, then reengaging the membrane in the next location in order to finish the peeling. After complete removal of the MP, Brilliant Peel Dual Dye was injected again. If there was no ERM seen on the inner surface of the retina, no further manipulation was performed. At the end of the surgery, only BSS was left inside the eye (see video, Supplementary materials 1, 2).

### Statistical analysis

The analysis of the data was performed by descriptive statistical analysis; ratio, mean ± standard deviation (SD), median and interquartile range (IQR), and range are presented. Normality of the continuous variables was tested using a histogram and the Shapiro-Wilk test. Paired sample *t*-test was used to compare the means of the two dependent, continuous, numerical variables when the normality assumption was satisfied; otherwise, Wilcoxon Signed Rank Test was used, when the normality assumption was not satisfied. The significance limit was set as *P* < 0.05. All statistical analyses were performed using the Statistical Package for STATA (Stata version 14; College Station, TX, USA) was used.

## Results

A total of fourteen eyes of fourteen patients were included in the study and further evaluated. There were six women and eight men patients. The mean age of the patients was 72.3 (range 59–84) years (Table 1).

**Table 1:** Characteristics of the studied patients.

**Number of patients**	**Age (years; mean (range))**	**Gender: female vs. male (ratio)**	**Pre-op BCVA (logMAR; median (IQR), range)**	**Post-op BCVA (logMAR; median (IQR), range)**	**Pre-op CRT (μm; mean ±SD)**	**Post-op CRT (μm; mean ±SD)**
14	72.3 (59–84)	6:8	0.7 (0.4–0.7) 0.3–1.0	0.15 (0.1–0.3) 0.0–1.0	489.71 ± 57.22	377.64 ± 41.05
**Number of patients**	**Eye (Right/Left) (ratio)**	**IOP Pre-op vs. Post-op (mmHg; mean ±SD)**	**Lens status Phakic/IOL at last follow-up (ratio)**	**Axial length** **(mm; mean ±SD)**	**Surgical time (min.; mean (range))**	**Previous cataract surgery (N)**
14	8:6	16.1 ± 2.8 vs. 14.3 ± 2.02	3:11	23.32 ± 0.76	19.43 (16.49–26.32)	4

BCVA, best corrected visual acuity; CRT, central retinal thickness; IOP, intraocular pressure; IOL, intraocular lens, IQR, interquartile range, SD, standard deviation.

**Figure 1:**
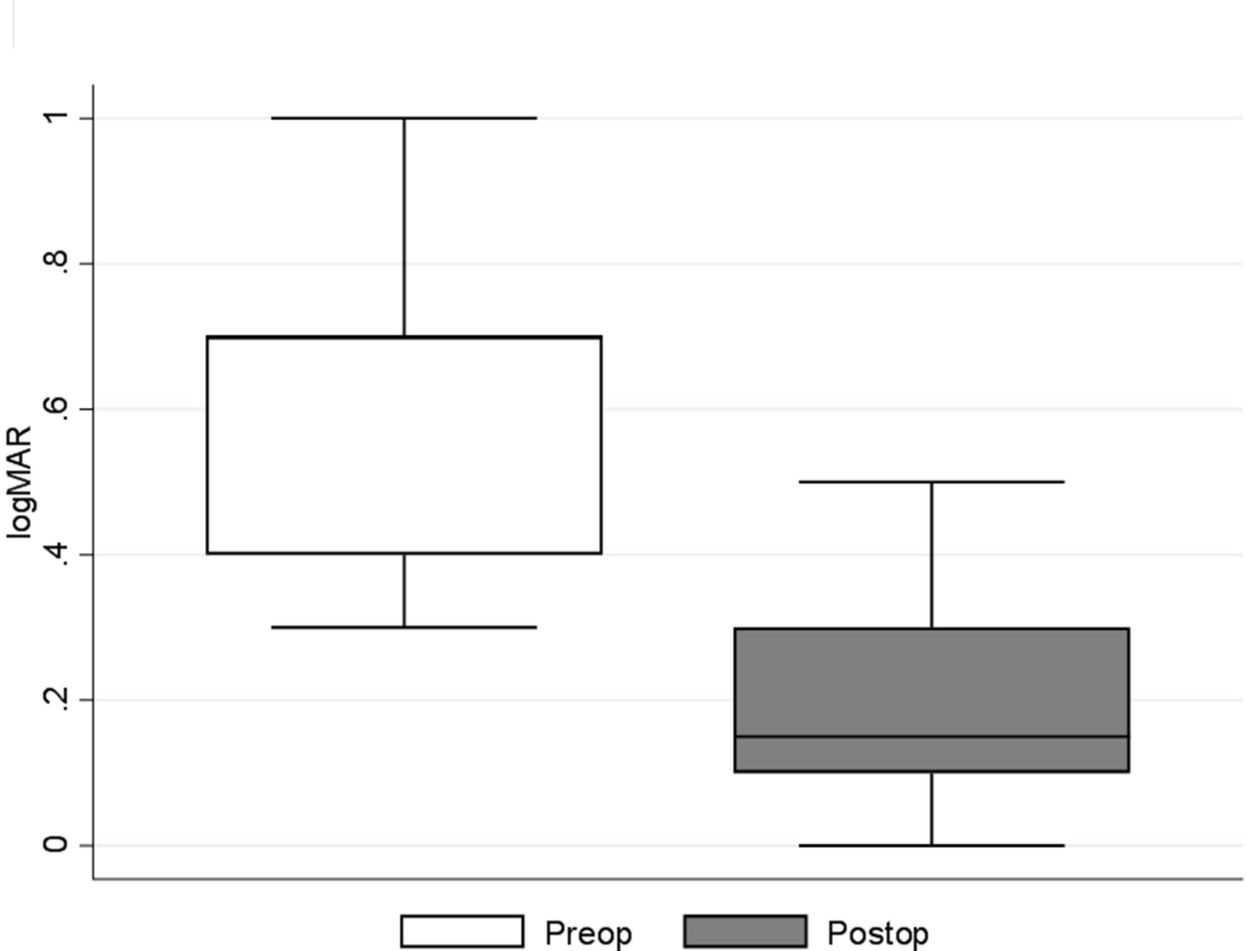
Median (IQR and range) of pre- and post-operative best corrected visual acuity (logMAR) results. Preop., pre-operative; Postop., post-operative.

**Figure 2:**
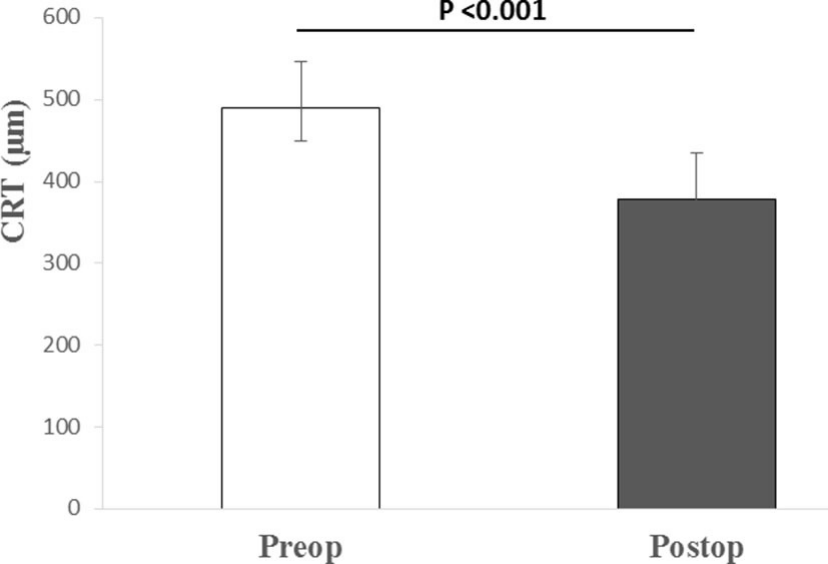
Median (IQR and range) of pre- and post-operative central retinal thickness. Preop., pre-operative; Postop., post-operative; CRT, central retinal thickness; μm, micrometers.

The median pre-operative BCVA was 0.7 (IQR: 0.4–0.7; range 0.3–1) on a logMAR scale. The mean ± SD of the pre-operative CRT was 489.71 ±57.22 μm. The patients had normal pre- and post-operative intraocular pressure, while the axial length was on average 23.32 ± 0.76 mm (Table 1). In one case the eye was amblyopic, while the systemic disease was present in 11 cases (most commonly arterial hypertension, hyperlipidemia, and diabetes mellitus without signs of diabetic retinopathy; least commonly: osteoporosis, benign prostatic hyperplasia, and cardiac disease). Combined phacoemulsification and vitrectomy were performed in five cases. In all cases, the MP was peeled off with the vitrectomy probe without the need for micro-forceps and without the need for additional procedures. Vitrectomies were finished without intraocular bleeding. All patients had follow-ups for at least 6 months. There were no intra- or post-operative complications registered. No cases of post-operative retinal detachment or glaucoma were registered. During the follow-up period, 2 patients had additional cataract surgery in the operated eye because of progressive nuclear sclerosis. The median post-operative BCVA 6 months after the surgery improved to 0.15 (IQR: 0.1–0.3; range: 0–1). Statistically significant median difference (*P* < 0.001) between the pre- and post-operative logMAR values was detected (Figure 1). The mean value of the post-operative CRT was 377.64 ± 41.05 μm. Statistically significant mean difference (*P* < 0.001) was detected between the pre- and post-operative CRT (Figure 2).

**Figure 3:**
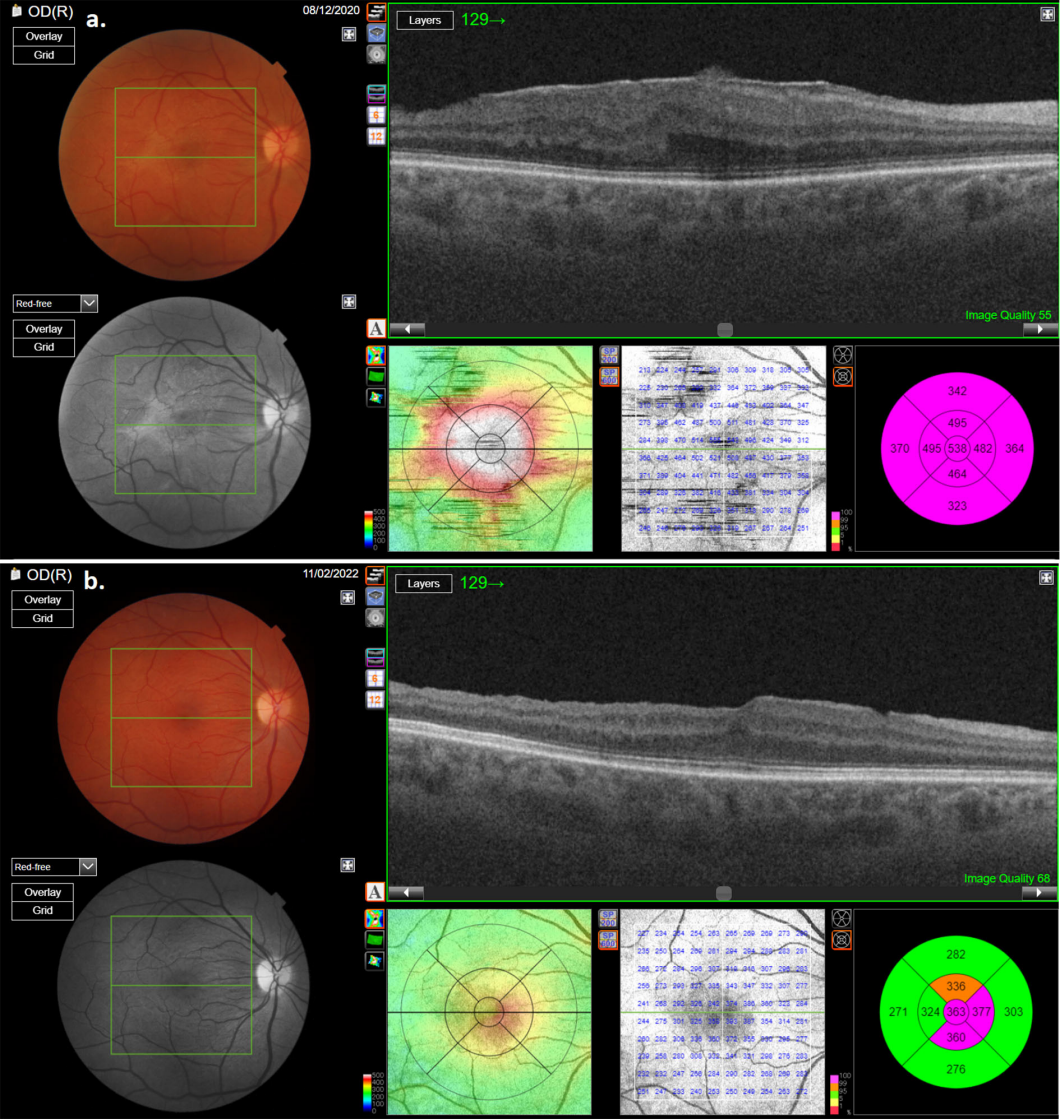
OCT scan of one of the patients with macular pucker: **(a)** pre-operative OCT scan showing thick macular pucker; **(b)** OCT scan of the same patient after surgery.

**Figure 4:**
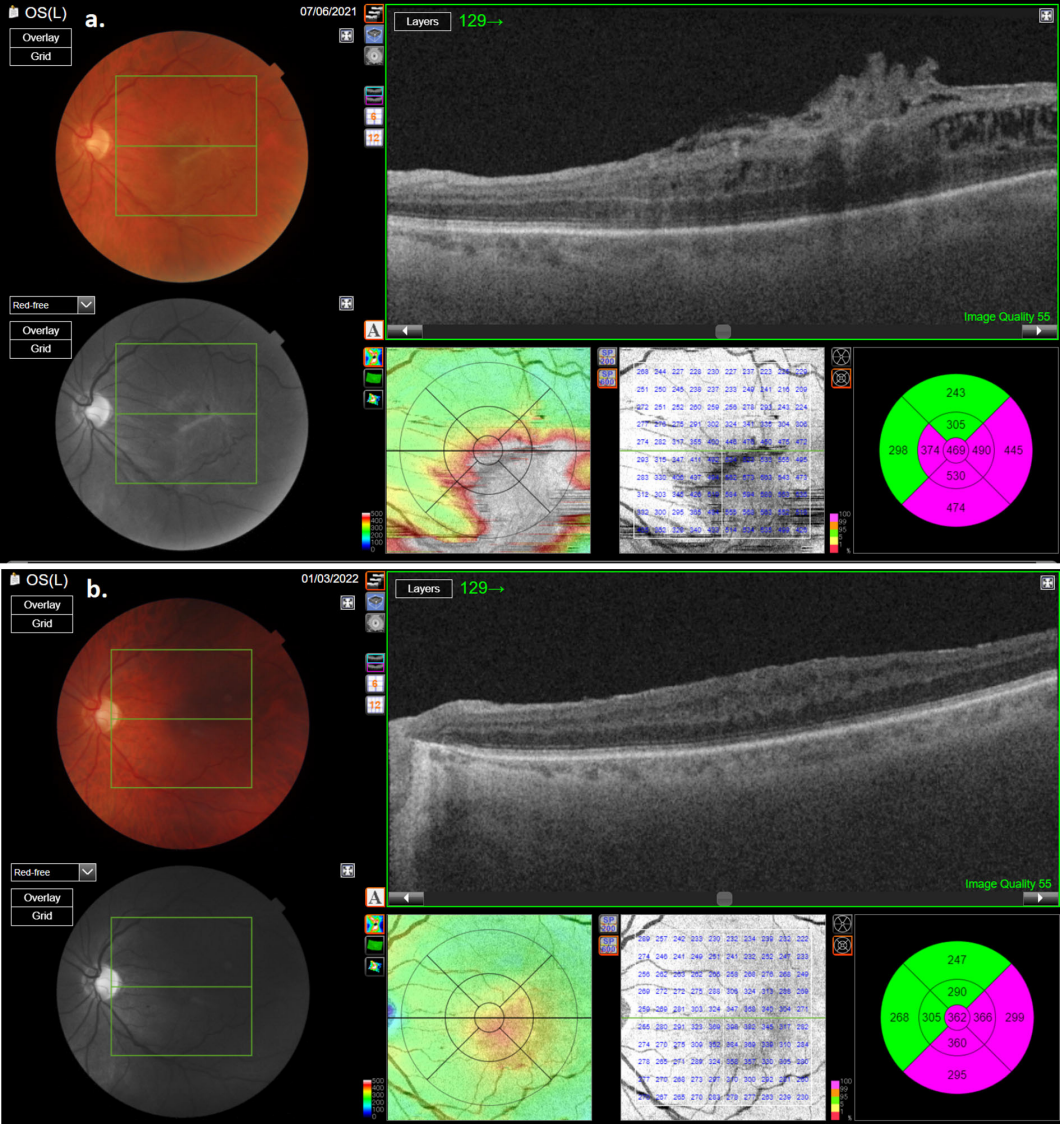
OCT scan of the second patient with macular pucker: **(a)** pre-operative OCT; **(b)** post-operative OCT showing reduction of retinal thickness.

During the post-operative follow-up period, no recurrence of ERM in any of the patients could be found (Figures 3, 4). In none of the cases did MH appearance occur. ELM and EZ continuity were post-operatively present in 13 out of 14 cases. SR was not detected in the study cases. RPE continuity was preserved in all patients (Figures 3, 4).

## Discussion

Surgery for ERM is one of the more common interventions in vitreoretinal surgery. As in any other surgical procedure, the main goal is to perform a complete removal of the pathologic tissue in as much as possible atraumatic manner. Many times, this is difficult. Even in the hands of experienced surgeons, iatrogenic damage by the different sharp tips of the instruments can happen (12).

In this pilot study, we present a novel surgical approach for MP removal without the need for extra instrumentation. The technique enables surgeons to simply engage the edge of the MP, then gradually increase the vacuum to achieve peeling of the membrane without the need for multiple pinches. Fewer pinches made reduce the possibility of iatrogenic damage to the central or peripheral retina.

In this case series, we have successfully removed complete MP without the need for forceps. Our experience with this approach is longer, but this is the first time we did a prospective analysis of the results of surgery. In all cases, the surgery was finished fast and without complications. Our impression is that the technique is simple and safe. In each case, the patient did well. BCVA improved in all cases. Morphological improvement was seen on OCT in all cases with reduced CRT.

None of the patients had IOP rise or developed glaucoma after the surgery. There are also previous reports that uncomplicated PPV for idiopathic ERM does not increase the risk of ocular hypertension or open-angle glaucoma development in long-term observations (13). However, Lin GC et al. found high IOP at baseline or during follow-up to be a significant factor associated with limited visual outcomes (14).

Surgery for ERM is generally considered safe and effective. Even in cases with very good pre-operative visual acuity, the treatment results in significant improvement in vision (15). Significant improvement of visual acuity after PPV for ERM has also been found in the analysis of Kishi et al. 3 months after the surgery, which was maintained over the 5-year follow-up period (16).

The ILM peeling procedure during surgery for ERM is being increasingly used by retinal surgeons. However, the effectiveness of the ILM peeling is uncertain and debatable. There are different and also contradictory reports on the outcomes of such peeling. In a meta-analysis by Chang et al., they found a more efficacious reduction of CRT in patients without ILM peel in comparison to patients with ERM and ILM peel. However, the long-term follow-up showed better functional improvement and a lower recurrence rate in patients with ILM peel (17). Another meta-analysis showed that patients without ILM peel had better visual improvement in the long-term than patients after both ERM and ILM peel (18). In a randomized multicenter clinical trial, Ripandelli et al. evaluated the retinal sensitivity, frequency of microscotomas, and other microperimetric parameters in patients with idiopathic MP—they found better outcomes in patients without ILM peel after a 12-month follow-up (19).

The fact is that ILM is a much more fragile, thinner, and less elastic structure compared to ERM, and our technique is probably less suitable for ILM alone. But in our opinion, this question remains to be answered in future studies and analyses. Since this is a pilot study and with the aim of eliminating potential dilemmas about the impact of ILM peeling on the final treatment outcomes, we decided to perform only ERM peeling.

In our hands, this technique works better with the 25-gauge system. It is possible to perform it with 23 and 27 gauge as well. But 23-gauge cutter has a wider opening, which can represent a higher chance of aspirating and accidentally engaging the retina. On the other hand, a 27-gauge cutter is more flexible and sometimes it can extensively bend during the peeling procedure.

Neither in this case series nor before we could not detect any complication related to the technique. We are aware that any surgical approach can go wrong and every surgical procedure can result in a bad outcome. However, in our opinion, at least in some cases, this approach may work well in reducing the costs for the management of MP, and also improving outcomes of the treatment.

## Conclusions

This technique could offer an alternative and safe approach for MP surgery without the need for extra instrumentation. In all cases, it was possible to remove the membrane in a safe, and cost-saving manner. In all cases, we achieved good functional and morphological outcomes.

## Data availability statement

The raw data supporting the conclusions of this article will be made available by the authors, without undue reservation.

## Ethics statement

The studies involving human participants were reviewed and approved by National Medical Ethics Committee of the Republic of Slovenia (Approval No. 91/05/11). The patients/participants provided their written informed consent to participate in this study.

## Author contributions

XL, BP, and GP did the literature review and wrote the manuscript. XL performed surgeries. BEP defined methodologies and performed statistical analysis. GP contributed to conception and design of the study. All authors contributed to the article and approved the final submitted version.

## Conflict of interest

The authors declare that the research was conducted in the absence of any commercial or financial relationships that could be construed as a potential conflict of interest.

## Publisher's note

All claims expressed in this article are solely those of the authors and do not necessarily represent those of their affiliated organizations, or those of the publisher, the editors and the reviewers. Any product that may be evaluated in this article, or claim that may be made by its manufacturer, is not guaranteed or endorsed by the publisher.

## Abbreviations

ERM: epiretinal membrane
OCT: optical coherence tomography
MP: macular pucker
ILM: inner limiting membrane
PPV: pars plana vitrectomy
BCVA: best corrected visual acuity
logMAR: logarithm of the minimum angle of resolution
CRT: central retinal thickness
MH: macular hole
ELM: external limiting membrane
EZ: ellipsoid zone
RPE: retinal pigment epithelium
SF: subretinal fluid.
